# Supplementary material for: Epidemiology and seasonal patterns of hospitalized dengue cases in Muscat Governorate, Oman (2022–2023): A retrospective study
Source: PLoS One. 2026 May 29;21(5):e0350734. doi: 10.1371/journal.pone.0350734 (PMC13221028; doi:10.1371/journal.pone.0350734)
Supplement: Attachment — Submitted filename: Reviewers Comments PONE-D-25-65079.docx [file pone.0350734.s001.docx]

**Overall Assessment (Reviewer Comment)**

**General Comment:**

This manuscript addresses an important and timely public health issue “**Dengue Epidemiology and Seasonality in Muscat Governorate, Oman**” using a relatively large, well-validated hospital-based dataset. The study is clearly written, methodologically sound, and adds local evidence to a region with limited published dengue data. However, several conceptual, methodological, and interpretative issues need clarification to strengthen scientific rigor, internal consistency, and generalizability. Most concerns relate to season definitions, interpretation of regression findings, representativeness of hospitalized cases, and overstatement of causal inferences.

**FINAL REVIEWER VERDICT**

**Recommendation: Major Revision**

**MAJOR COMMENTS (Require Substantive Revision)**

**1. Inconsistency and Conceptual Ambiguity in Season Definition**

The manuscript defines the dry season as **May–October** and the wet season as **November–April** (Methods, line 187). However, **peak dengue cases are repeatedly described as occurring in April–May**, which straddles both seasons. This creates conceptual ambiguity and weakens the interpretation of “wet-season dengue risk.”

Clarify the seasonal categorization using meteorological data or authoritative local climate references.

Consider sensitivity analysis using alternative seasonal cutoffs (e.g., pre-monsoon, monsoon, post-monsoon).

**2. Interpretation of Logistic Regression Findings**

The multivariable analysis identifies hospital of admission and PCR positivity as predictors of wet-season dengue. However, these variables are health-system and diagnostic factors, not epidemiological risk factors for dengue transmission.

Concern:

The manuscript occasionally implies epidemiological or environmental significance, which may mislead readers.

Therefore, reframe these findings consistently as healthcare system–related associations, not determinants of transmission.

Clarify that PCR positivity likely reflects testing timing and clinical suspicion rather than increased incidence.

Avoid causal language when discussing adjusted odds ratios.

**3. Limited External Validity and Selection Bias**

The study includes only hospitalized patients from two tertiary hospitals, representing moderate-to-severe cases. This substantially limits generalizability to community level dengue epidemiology.

Recommendation:

Emphasize throughout (Abstract, Discussion, Conclusion) that findings apply primarily to hospitalized dengue cases.

Avoid statements suggesting population-level dengue risk or overall incidence patterns without outpatient or community data.

**4. Mortality Rate Needs Contextualization**

The reported mortality rate (3.8%) appears high compared to many dengue-endemic settings.

Recommendation:

Provide here contextual comparison with regional or hospital-based mortality rates.

Clarify whether deaths were directly attributable to dengue or involved significant comorbidities.

Consider reporting case fatality rate stratified by severity.

**5. Overextension of Public Health Implications**

While recommendations for vector control and preparedness are appropriate, the study lacks direct entomological or climatic data, limiting the strength of policy implications.

Recommendation:

Temper conclusions to align with the study’s descriptive and hospital-based nature.

Explicitly state that vector control recommendations are supported by existing literature, not direct measurements from this study.

MINOR COMMENTS (Editorial / Clarity Issues)

**1. Title**

The title contains a typographical error: “Retrospective Stud” → “Retrospective Study”.

Also consider specifying hospitalized cases for accuracy.

Suggested Revision:

“Epidemiology and Seasonal Patterns of Hospitalized Dengue Fever Cases in Muscat Governorate, Oman (2022–2023): A Retrospective Study”

**2. Abstract – Results**

Percentages are sometimes presented without clear denominators (e.g., Wilayat distribution). Consider briefly clarifying the population base.

**3. Abstract – Conclusions**

The phrase “higher wet-season risk” may be misleading given that total cases were higher in the dry season.

Suggested Revision:

Replace with: “distinct seasonal and geographic clustering” rather than “higher wet-season risk.”

**4. Methods – Sampling Technique**

The justification for convenience sampling is acceptable, but the phrase “reduce selection bias” is inaccurate.

Suggested Revision:

Replace with: “to maximize sample size, acknowledging potential selection bias inherent to retrospective hospital-based data.”

**5. Tables**

Tables 2 and 3 contain some inconsistencies in spacing and labeling (e.g., “Auto immune” vs. “Autoimmune”). Ensure uniform terminology and formatting across all tables.

**6. Figures**

Figures are referenced appropriately, but captions could be more descriptive, particularly Figure 1, by stating whether counts represent admissions or cases.

**SECTION-BY-SECTION ANNOTATED COMMENTS**

**Introduction**

The introduction is comprehensive and well referenced but could be more concise. Some global statistics (e.g., global burden, CDC estimates) are repeated or overly detailed relative to the local study aim.

Suggestion:

Streamline global burden discussion and sharpen the knowledge gap specific to Oman.

**Materials and Methods**

Data validation procedures are well described and strengthen credibility. However, absence of meteorological variables limits seasonal inference.

Suggestion: Explicitly state that seasonality is inferred indirectly.

**Results**

Results are clearly presented and tables are informative. However, p-values are sometimes emphasized without sufficient discussion of clinical relevance.

**Discussion**

The discussion is well structured and thoughtfully interprets findings. Strength lies in distinguishing health system effects from epidemiological drivers.

This distinction should be made more consistently throughout.

**References**

Need reference formatting according to journal guidelines. Add more updated references of 2026.

**Limitations**

Limitations are appropriately acknowledged but could include:

1. Misclassification bias due to season definition
2. Potential referral bias between hospitals
3. Conclusion

Comment:

The conclusion is balanced but could be tightened to avoid restating results already discussed.

**FINAL REVIEWER VERDICT**

**Recommendation: Major Revision**

The manuscript is publishable after addressing conceptual clarity on seasonality, refining interpretation of regression findings, and moderating causal and policy claims.
