# Supplementary material for: Epidemiology and seasonal patterns of hospitalized dengue cases in Muscat Governorate, Oman (2022–2023): A retrospective study
Source: PLoS One. 2026 May 29;21(5):e0350734. doi: 10.1371/journal.pone.0350734 (PMC13221028; doi:10.1371/journal.pone.0350734)
Supplement: Attachment — Submitted filename: Response to reviewer_ 7th April 2026.docx [file pone.0350734.s003.docx]

**7^th^ April 2026**

**Author Response to Reviewers’ Comments**

Dear Editors and Reviewers,

We would like to express our sincere appreciation for the time and effort you have devoted to the evaluation of our manuscript entitled ***“Epidemiology and Seasonal Patterns of Dengue in Muscat Governorate, Oman (2022–2023): A Retrospective Study”* (Manuscript ID: PONE-D-25-65079).**

We are grateful for the insightful and constructive comments provided, which have greatly contributed to improving the quality and clarity of our work. In response, we have carefully revised the manuscript and addressed all comments in a detailed, point-by-point manner, as outlined below.

All revisions have been incorporated into the manuscript, and both clean and track-changed versions have been submitted for your review.

Thank you for your consideration.

**MAJOR COMMENTS**

**1. Inconsistency and Conceptual Ambiguity in Season Definition**

**Reviewer Comment:**
Season definition (dry: May–October; wet: November–April) conflicts with peak dengue cases (April–May), creating ambiguity. Suggest clarification and sensitivity analysis.

**Author Response:**
Thank you for your feedback. We appreciate this important observation. We agree that the peak dengue months (April–May) overlap the seasonal transition period, which may create interpretive ambiguity.

To address this:

1. We have clarified in the Methods section that seasonal classification is based on official Muscat climatic rainfall patterns (Oman Meteorology Directorate data).
2. We have explicitly added a statement acknowledging that April–May represents a transitional period between wet and dry seasons, which may influence peak transmission patterns.
3. We have emphasized in the Discussion and Limitations that seasonality was inferred indirectly and not based on entomological or meteorological measurements at the patient level.
4. We acknowledge that a formal sensitivity analysis using alternative seasonal cut-offs (e.g., pre-monsoon/post-monsoon) is valuable; however, this was not feasible due to the predefined surveillance classification framework used in national reporting datasets.

**Manuscript Changes: (in clean copy)**

1. Methods (Exclusion Criteria) revised for clarity (Page no. 7, Line no. 161-165)
2. Discussion expanded (Seasonal Trends section) (Page no. 16 , Line no. 303-307)
3. Limitations updated to reflect classification constraint (Page no. 20, Line no.388-390)

**2. Interpretation of Logistic Regression Findings**

**Reviewer Comment:**
Hospital of admission and PCR positivity are health-system factors, not transmission determinants. Avoid causal interpretation.

**Author Response:**
Thank you for your feedback. We fully agree with this interpretation. The manuscript has been revised to ensure that:

1. Hospital of admission and PCR positivity are now consistently described as health-system and diagnostic-related variables, not epidemiological determinants.
2. We have explicitly removed any wording suggesting causality.
3. We clarified that PCR positivity likely reflects testing timing, clinical suspicion, and diagnostic workflow differences, rather than seasonal transmission intensity.

**Manuscript Changes:**

4. Results section revised to avoid causal wording (Page no.10, Line no. 203-204)
5. Discussion strengthened to emphasize health-system interpretation (Page no.19, Line no.369-371)
6. Conclusion modified to remove implied transmission causality (Page no.21)

**3. Limited External Validity and Selection Bias**

**Reviewer Comment:**
Hospitalized cases limit generalizability; emphasize study population constraints.

**Author Response:**
Thank you for your feedback. We agree with this important limitation. We have strengthened the manuscript to clearly state that:

1. Findings apply only to hospitalized dengue patients, representing moderate to severe disease.
2. Results cannot be generalized to community-level dengue incidence or mild outpatient cases.
3. This limitation is now emphasized in the Abstract, Discussion, and Conclusion.

**Manuscript Changes:**

1. Abstract revised for scope clarity (Page no.2)
2. Discussion expanded on selection bias (Page no.15, Line no.284-287)
3. Conclusion modified to avoid population-level inference (Page no.21).

**4. Mortality Rate Needs Contextualization**

**Reviewer Comment:**
Mortality rate (3.8%) requires comparison and clarification of attribution.

**Author Response:**
Thank you for your feedback. We appreciate this suggestion. We have now:

1. Added contextual comparison noting that mortality varies across hospital-based dengue studies in the region.
2. Clarified that deaths represent all-cause in-hospital mortality among confirmed dengue cases, and may include contributions from comorbid conditions.
3. Added recommendation for future stratification by disease severity and comorbidity status.

**Manuscript Changes:**

1. Discussion expanded (Clinical Characteristics section) (Page no.19, Line no.360-361)
2. Limitations updated (Page no.20, Line no.391-392)
3. Conclusion tempered (Page no.20, Line no.397-398)

**5. Overextension of Public Health Implications**

**Reviewer Comment:**
Policy recommendations should be tempered due to lack of entomological/climatic data.

**Author Response:**
Thank you for your feedback. We agree and have revised the manuscript accordingly:

1. All policy statements have been reframed as supportive recommendations based on existing literature, not direct study evidence.
2. We removed overly strong causal language linking findings to vector control effectiveness.
3. We emphasized that recommendations are contextual and hypothesis-generating.

**Manuscript Changes:**

1. Discussion revised (Public Health Implications section) (Page no.16-17, Line no.309-316)
2. Conclusion softened (Page no.20, Line no.403-405)
3. Added explicit limitation on lack of vector/climate data (Page no.20, Line no. 388-390)

**MINOR COMMENTS**

**1. Title Correction**

**Response:**
Thank you for your suggestion. The typographical error has been corrected, and the title has been refined to improve specificity.

**Revised Title:**
*Epidemiology and Seasonal Patterns of Hospitalized Dengue Fever Cases in Muscat Governorate, Oman (2022–2023): A Retrospective Study*

**2. Abstract – Results Clarity**

**Response:**
Thank you for your feedback. We have clarified denominators and ensured all percentages are clearly referenced to the study population (n=345).

**3. Abstract – Conclusion Wording**

**Response:**
We agree that “higher wet-season risk” may be misleading.

**Revision made:**
Thank you for your feedback. Replaced with: *“distinct seasonal and geographic clustering”*

**4. Methods – Sampling Technique**

**Response:**
Thank you for your feedback. We corrected the wording as suggested.

**Revision:**
Replaced “to reduce selection bias” with:
*“to maximize sample size, acknowledging potential selection bias inherent to retrospective hospital-based data.”*

**5. Tables – Formatting Issues**

**Response:**
Thank you for your feedback. All tables have been revised for:

1. Consistent terminology (e.g., “Autoimmune” standardized) (Page no.13, Line no.241-243)
2. Correct spacing and alignment (Page no.13, Line no.241-243)
3. Uniform labeling across tables (Page no.13, Line no.241-243)

**6. Figures – Caption Clarity**

**Response:**
Thank you for your feedback. Figure captions have been improved to clearly specify:

1. Data represents admissions (already mentioned: Monthly Distribution of Admitted Dengue Fever Patients during 2022-2023 in The Muscat Governorate (SQUH and RH)

**SECTION-BY-SECTION COMMENTS**

**Introduction**

**Response:**
Thank you for your feedback. We have streamlined global burden data and sharpened the Oman-specific research gap to improve focus and conciseness. (Page. 4, Line 108).

**Methods**

**Response:**
Thank you for your feedback. We clarified that seasonality is inferred indirectly and not based on direct meteorological measurements. (Page 7, line 164).

**Results**

**Response:**
Thank you for your feedback. We reduced emphasis on statistical significance alone and ensured clearer interpretation alongside clinical relevance. (Page 14, Line 256).

**Discussion**

**Response:**
Thank you for your feedback. We strengthened consistency in distinguishing:

1. Health-system effects
2. Epidemiological transmission drivers

**Limitations**

**Response:**
Thank you for your feedback. We added:

1. Referral bias between hospitals (Page no. 21, Line no. 391)
2. Absence of entomological/climatic data (Page no.20, Line no. 388)

**Conclusion**

**Response:**
Thank you for your feedback. The conclusion has been tightened to avoid repetition and overinterpretation of results. (Page. 21, Line 393)

**References: (Page.23, Line 436)**

**Updated Reference:**

**no. 1 (line 437, page.23):** Ministry of Health. Oman National Guidelines for Clinical Management of Dengue Disease [Internet]. Ministry of Health, Sultanate of Oman; 2024. Available from: https://moh.gov.om/en/health-promotion/health-awareness/dengue-fever/

**no. 26 (line 515, page.25):** Prabhat Pandey MMP. RESEARCH METHODOLOGY: TOOLS AND TECHNIQUES [Internet]. Bridge Center; 2015. Available from: <https://www.euacademic.org/BookUpload/9.pdf>

**Added References:**

**no. 3 (line 443, page.23):** He S, Fan D, Guo Y, Guan Y, Sheng Z, Gao N, et al. Current status of dengue fever epidemics and vaccine development. Virologica Sinica. 2026 Feb;41(1):1–9. doi:10.1016/j.virs.2026.01.001

**no. 4 (line 446, page.23):** Shoushtari M, Bakhshi H, Salehi-Vaziri M, Zaim M, Enayati A, Pouriayevali MH, et al. Distribution of Aedes aegypti and Aedes albopictus, and the current situation of dengue fever and chikungunya in Iran and neighboring countries: a review study. Gebeyehu DG, editor. PLoS Negl Trop Dis. 2026 Feb 11;20(2):e0013965. doi:10.1371/journal.pntd.0013965

**No. 4 (line446, page. 23):** Shoushtari M, Bakhshi H, Salehi-Vaziri M, Zaim M, Enayati A, Pouriayevali MH, et al. Distribution of Aedes aegypti and Aedes albopictus, and the current situation of dengue fever and chikungunya in Iran and neighboring countries: a review study. Gebeyehu DG, editor. PLoS Negl Trop Dis. 2026 Feb 11;20(2):e0013965. doi:10.1371/journal.pntd.0013965

**No. 14 (line 483, page. 24):** Oman Metro Area Population 1950-2025. Muscat, Oman Metro Area Population 1950-2025. 2025. doi:https://www.macrotrends.net/global-metrics/cities/21922/muscat/population

**No. 17 (line 491, page. 24):** Al Kishri W, Baksh N. Rainfall Forecasting in Muscat Governorate Using Artificial Neural Networks and Hybrid Modeling Approaches. SUJEITI. 2025 Jul 12;1(2). doi:10.69983/SUJEITI/1222

**No. 19 (line 497, page. 25):** Al-Manji A, Wirayuda AAB, Al Wahaibi A, Al-Azri M, Chan MF. Investigating the Determinants of Dengue Outbreak in Oman: A Study in Seeb. J Epidemiol Glob Health. 2024 Nov 4;14(4):1464–75. doi:10.1007/s44197-024-00324-3

**FINAL NOTE**

We thank the reviewers again for their insightful feedback. The revisions have significantly improved the clarity, scientific rigor, and interpretative balance of the manuscript.

Sincerely,
**Ms. Ibtisam Khalifa Al Maskari**

**First author, on behalf of all authors**
